# Brassinosteroids Improve Quality of Summer Tea (*Camellia sinensis* L.) by Balancing Biosynthesis of Polyphenols and Amino Acids

**DOI:** 10.3389/fpls.2016.01304

**Published:** 2016-08-30

**Authors:** Xin Li, Golam J. Ahammed, Zhi-Xin Li, Lan Zhang, Ji-Peng Wei, Chen Shen, Peng Yan, Li-Ping Zhang, Wen-Yan Han

**Affiliations:** ^1^Key Laboratory of Tea Biology and Resources Utilization, Ministry of Agriculture, Tea Research Institute, Chinese Academy of Agricultural SciencesHangzhou, China; ^2^Department of Horticulture, Zhejiang UniversityHangzhou, China; ^3^Graduate School of Chinese Academy of Agricultural SciencesBeijing, China

**Keywords:** 24-epibrassinolide, amino acids, photosynthesis, polyphenol, secondary metabolism, summer tea, tea quality

## Abstract

Summer grown green tea is less popular due to bitterness and high astringency, which are attributed to high levels of tea polyphenols (TP) and low levels of amino acids (AA) in tea leaves (*Camellia sinensis* L.). Brassinosteroids (BRs), a group of steroidal plant hormones can regulate primary and secondary metabolism in a range of plant species under both normal and stress conditions. However, specific effects of BRs on the photosynthesis of tea plants and the quality of summer green tea are largely unknown. Here we show that 24-epibrassinolide (EBR), a bioactive BR, promoted photosynthesis in tea plants in a concentration-dependent manner. Stimulation in photosynthesis by EBR resulted in an increased summer tea yield. Although all tested concentrations (0.01, 0.05, 0.1, 0.5, and 1.0 ppm) of EBR increased concentrations of TP and AA, a moderate concentration (0.5 ppm) caused the highest decrease in TP to AA ratio, an important feature of quality tea. Time-course analysis using 0.5 ppm EBR as foliar spray revealed that TP or AA concentration increased as early as 3 h after EBR application, reaching the highest peak at 24 h and that remained more or less stable. Importantly, such changes in TP and AA concentration by EBR resulted in a remarkably decreased but stable TP to AA ratio at 24 h and onward. Furthermore, concentrations of catechins and theanine increased, while that of caffeine remained unaltered following treatment with EBR. EBR improved activity of phenylalanine ammonia-lyase (PAL) and glutamine: 2-oxoglutarate aminotransferase (GOGAT) enzymes involved in catechins and theanine biosynthesis, respectively. Transcript analysis revealed that transcript levels of *CsPAL* and *CsGS* peaked as early as 6 h, while that of *CsGOGAT* peaked at 12 h following application of EBR, implying that EBR increased the concentration of TP and AA by inducing their biosynthesis. These results suggest a positive role of BR in enhancing green tea quality, which might have potential implication in improving quality of summer tea.

## Introduction

Green tea is a widely consumed beverage both in the east and the west (Tounekti et al., 2013). It is an unfermented tea, produced from the young leaves (usually the apical bud and two leaves below the bud) of *Camellia sinensis* L., where major portion of tea polyphenols (TP) is kept unoxidized during tea processing. TP are flavonoids, synthesized through secondary metabolic pathways such as shikimate pathway, phenylpropanoid pathway, and flavonoid pathway (Tounekti et al., 2013; Verma and Shukla, 2015). TP have numerous health benefits including prevention of cancer, cardiovascular, neurodegenerative and other oxidative stress-related diseases (Bordoni et al., 2002). Furthermore, TP are considered as one of the important determinants of tea quality as they impart astringency. Nonetheless, too high TP concentrations make tea infusion too bitter (Liang et al., 1996). In addition to TP, concentration of total free amino acids (AA) is a major factor determining green tea quality. AA are responsible for the freshness and mellowness of green tea. However, the quality of green tea not only depends on concentration of TP and AA, rather largely on TP to AA ratio. In general, the value of TP to AA ratio is inversely correlated with green tea quality, where a lower TP to AA ratio makes tea more brisk and mellow, but less bitter in taste (Liang et al., 1996; Wang et al., 2011; Xu et al., 2012; Tounekti et al., 2013).

It is well known that the levels of TP and AA are influenced by many factors, including harvest season. The TP to AA ratio varies mainly due to variations in the day length, rainfall, sunlight, and/or temperature that are distinct in each season (Tounekti et al., 2013). Based on the harvest seasons, Chinese green tea can be divided into three types such as “spring tea,” “summer tea,” and “autumn tea” (Xu et al., 2012). Spring teas, which are harvested in cool months (before late May) comprise a higher level of AA but a moderate level of TP with an optimum TP to AA ratio. On the other hand, summer teas which are harvested in warmer months (between early June and early July) possess a lower level of AA and a higher level of TP and TP to AA ratio. Therefore, the spring teas taste better than the summer teas and autumn teas. Such metabolic response to season is possibly conserved among all genotypes of *C. sinensis* L. (Wang et al., 2011). This implies that the cultivation of the best quality tea is limited to a short period (spring season) of a year (Xu et al., 2012). Therefore, development of strategies for improving summer tea quality is one of the cutting edge issues in the tea research.

Phytohormones are endogenous messenger molecules that regulate various aspects of plant growth, development, and responses to stress (Ahammed et al., 2014). A prior study showed that exogenous application of phytohormone gibberellins (GA) could not only increase AA concentration but also decrease TP concentration, leading to a decreased TP to AA ratio in green tea (Liang et al., 1996). Exogenous application of methyl jasmonate (MeJA) improves the aroma quality of black tea (Shi et al., 2014). Furthermore, individual treatment with GA and abscisic acid alters the concentration of catechins (flavan-3-ols, major bioactive TP) as well as transcript levels of its biosynthetic genes such as *CsPAL*, *CsC4H*, *Cs4CL*, *CsF3H*, and CsANR (Singh et al., 2009; Rani et al., 2012). All these reports clearly indicate that phytohormones are potentially involved in controlling the quality of tea by modulating secondary metabolism in tea plant.

Brassinosteroids (BRs), a group of steroidal plant hormones, play critical roles in the regulation of both primary and secondary metabolism in a range of plant species (Ahammed et al., 2012, 2014; Çoban and Göktürk Baydar, 2016). For instance, exogenous application of 24-epibrassinolide (EBR, a bioactive BR) promotes CO_2_ assimilation capacity by enhancing ribulose-1,5-bisphosphate carboxylase/oxygenase (RuBisCO) carboxylation rate, activities of RuBisCO activase and fructose-1,6-bisphosphatase in cucumber plants (Yu et al., 2004; Jiang et al., 2012a). BRs regulate Benson–Calvin cycle and sugar metabolism via redox signaling, which eventually increases the photosynthetic potential and biomass accumulation in plants (Jiang et al., 2012c). Exogenous application of EBR could increase the activity of secondary metabolism-related enzymes such as phenylalanine ammonia-lyase (PAL, the first enzyme involved in flavonoid biosynthesis), resulting in an increased concentration of phenols and flavonoids in tomato roots and *Vitis vinifera* grape berry (Ahammed et al., 2012; Xi et al., 2013). In peppermint (*Mentha piperita* L.), foliar application of 0.5 mg l^-1^ EBR could increase total phenolic content around twofold in leaves compared with that of control plants (Çoban and Göktürk Baydar, 2016). Moreover, combined application of EBR and MeJA could sharply induce concentration of secondary metabolites in sweet basil (*Ocimum basilicum* L.; Koca and Karaman, 2015). Although BRs were identified in tea leaves (*Thea sinensis*) about 35 years ago, soon after the discovery of brassinolide in the pollen of *Brassica napus* (Morishita et al., 1983), no literature is currently available for the effects of BRs on the secondary metabolism in tea plants.

Notably, characterization of novel microRNA (miRNA) in tea suggests that miRNA-mediated BR signaling might play an important role in regulating developmental and seasonal variations in tea (Mohanpuria and Yadav, 2012). A recent study showed that amiRNA designated as cs-miR414 is profoundly expressed in dormant bud of tea compared with that in active bud, and cs-miR414 targets mRNAs that are involved in maintaining the endogenous concentration of BRs and its homeostasis, implying that BRs level is critical for bud dormancy in tea (Jeyaraj et al., 2014). Based on these recent reports and the role of BRs in secondary metabolism in various plant species, we hypothesized that BRs might influence the concentration of TP and AA in tea leaves, and thus the quality of green tea. To elucidate specific effects of BRs on the photosynthesis of tea plants and the quality of green tea, we analyzed the CO_2_ assimilation rate in tea plants after application of EBR. In addition, we examined effects of EBR on the quality of green tea by measuring concentration and ratio of TP and AA as well as concentration of catechins, theanine and caffeine. Our results suggest that BRs have a significant stimulatory effect on photosynthesis and quality of green tea.

## Materials and Methods

### Plant Material and Growth Conditions

For the current experiment, widely cultivated tea (*C. sinensis* L.) cultivar in China namely Longjing 43 was chosen. The experiment was conducted during summer (in the month of July), when mean maximum and minimum temperatures were 37.4 ± 0.55°C and 28.2 ± 1.3°C, respectively and relative humidity: 42.0 ± 3.2% at tea garden of the Tea Research Institute, Chinese Academy of Agricultural Sciences, Hangzhou, Zhejiang province, China (longitude 120°10′E and latitude 30°14′N, 16 m above sea level). Foliar portion of tea bushes was sprayed with freshly prepared 24-EBR (Sigma-Aldrich, St. Louis, MO, USA). A graded levels of working solution of EBR (0.01, 0.05, 0.1, 0.5, and 1.0 ppm) was prepared by dissolving solute in ethanol followed by dilution with MilliQ water [ethanol:water (v/v) = 1:10000]. Control (CK) tea bushes were simultaneously sprayed with MilliQ water containing same ratio of ethanol. Each treatment comprises four replicates, while each replicate represents an area of 10 m^2^ consisting of 20 tea bushes.

### Photosynthesis Measurement

Twenty-four hours after EBR application, net CO_2_ assimilation rate (Pn) was measured on 3^rd^ fully expanded leaves using an open-flow infrared gas analyzer adapted with light and temperature control systems (Li-COR 6400, Lincoln, NE, USA) in 12 tea bushes under each treatment. The measurement was performed within the time period from 8:00 am to 11:00 am maintaining the air temperature, relative humidity, CO_2_ concentration and photosynthetic photon flux density (PPFD) at 25°C, 80%, 400 μmol mol^-1^ and 800 μmol m^-2^ s^-1^, respectively.

### Quantification of Tea Polyphenols and Total Free Amino Acids

Total TP was extracted and determined spectrophotometrically according to the standard method established by the International Organization for Standardization (ISO) 14502-1 using gallic acid as standard (ISO 14502-1, 2005; Anesini et al., 2008). In brief, the diluted sample extract (1.0 ml) was transferred to tubes in duplicate, where each tube contained 5.0 ml of a 1/10 dilution of Folin–Ciocalteu’s reagent in water. Afterward, 4.0 ml sodium carbonate solution (7.5% w/v) was added into each tube. The tubes were kept at room temperature for 60 min before absorbance at 765 nm was measured against water.

Total AA from tea leaf sample (0.5 g) were extracted in 80% ethanol at 80°C. Following evaporation, dried samples were dissolved in 0.02 N HCl. AA, separated by cation-exchange chromatography, were subjected to postcolumn reaction with ninhydrin reagent and detected spectrophotometrically as described previously elsewhere (Chen et al., 2010).

### Determination of Catechins, Caffeine, and Individual Amino Acids

The concentrations of caffeine and catechins in the extract were determined with a high-performance liquid chromatography (HPLC) system (Waters 590; Waters Corp., Milford, MA, USA) equipped with a Hypersil ODS2 C18 column (5 ml, 4.6 mm × 250 mm, 35°C) at 280 nm as previously described (Su et al., 2003). Solvents A (2% acetic acid) and B (acetonitrile) were run in linear gradients with A decreasing from 93 to 55% within 20 min and maintained for 5 min, thereafter at a rate of 1.4 ml min^-1^. Concentrations of caffeine and catechins were quantified by their peak areas against those of standards prepared from authentic compounds. Individual AA (theanine) were measured by using an automatic AA analyzer (Hitachi L-8900, Japan). Five milliliters of tea extract was added with 5 ml of sulfosalicylic acid and the mixture was centrifuged at 13,000 rpm for 5 min to facilitate the reaction. The mixture was filtered through a 0.20 μm nylon filter membrane and run in the AA analyzer (Wang et al., 2006).

### Determination of PAL and GOGAT Activities

For PAL activity assay, 0.3 g tea leaf sample was homogenized in 3 ml 50 mM potassium phosphate buffer (pH 8.8, containing 2 mM ethylenediaminetetraacetic acid (EDTA), 2% polyvinyl polypyrrolidone (PVPP), and 0.1% mercaptoethanol). The homogenate was centrifuged at 15,000 rpm for 20 min at 4°C and the supernatant fractions were collected as a crude enzyme extract. PAL activity was estimated with L-phenylalanine as substrate (Zheng et al., 2005).

The extract for glutamine: 2-oxoglutarate aminotransferase (GOGAT) measurement was obtained by grinding 0.3 g frozen leaf sample in 2 ml 25 mM Tris–HCl buffer (pH 7.8) containing 1 mM MgCl_2_, 1 μM β-mercaptoethanol, 1 mM EDTA, and 1% (w/v) PVPP. For assays, the extract was centrifuged at 15,000 rpm for 20 min, and the GOGAT activities were measured in the supernatant as described by Suzuki et al. (2001).

### RNA Isolation and Transcript Analysis

Total RNA from tea leaves was prepared using TRIzol reagent (Invitrogen, Carlsbad, CA, USA) according to the manufacturer’s instruction. A purifying column was used to remove genomic DNA from RNA samples. Reverse transcription was done using Superscript II (Invitrogen) following the manufacturer’s protocol. The primers used for transcript analysis have been listed in Supplementary Table S2. qRT-PCR analysis was carried out using the StepOnePlus Real-Time PCR system (Applied Biosystems, Foster City, CA, USA) with Power SYBR Green PCR Master Mix (Applied Biosystems). Transcript abundance was normalized to *actin*, and relative gene expression was calculated following formulae of Livak and Schmittgen (2001). qRT-PCR conditions consisted of denaturation at 95°C for 3 min, followed by 40 cycles of denaturation at 95°C for 30 s, annealing at 58°C for 30 s and extension at 72°C for 30 s.

### Statistical Analysis

All data were analyzed using the statistical software SAS 8.1 (SAS Institute Inc., Cary, NC, USA). Following variance analysis, an analysis of least significant difference (*P* < 0.05) among means was performed by the Duncan’s multiple range test.

## Results

### Photosynthetic Response of Tea Plants to Exogenous EBR Is Concentration Dependent

**FIGURE 1:**
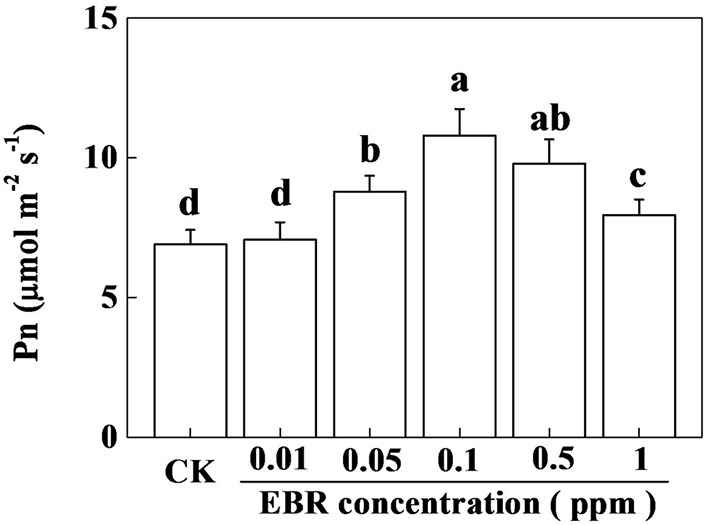
**Effect of different concentrations of 24-epibrassinolide (EBR) on net photosynthetic rate (Pn) in tea plants.** Tea bushes were sprayed with graded levels of EBR (0.01, 0.05, 0.1, 0.5, and 1.0 ppm) solution. Control (CK) tea bushes were simultaneously sprayed with MilliQ water containing same ratio of ethanol used to dissolve EBR. Twenty-four hours after EBR application, Pn was measured on third fully expanded leaves using an open-flow infrared gas analyzer adapted with light and temperature control systems (Li-COR 6400, Lincoln, NE, USA). The results are expressed as the mean values ± SD. Mean denoted by different letters indicate significant differences between the treatments (*P* < 0.05).

Several lines of evidence suggest that plant response to BR is highly concentration specific (Jiang et al., 2012b; Ahammed et al., 2014). To understand the photosynthetic response of tea plants to BR, we treated foliar portion of tea plants with various concentrations of EBR ranging from 0.01 to 1.0 ppm. Twenty-four hours after application of EBR, net photosynthetic rate (Pn) was measured in control (CK) and EBR-treated tea plants. As shown in **Figure 1**, the lowest concentration of EBR (0.01) had no effect on Pn, while 0.05 ppm EBR caused a significant increase in Pn, leading to a gradual increase in Pn up to 0.1 ppm EBR. After reaching the highest Pn value at 0.1 ppm EBR, which was increased by 56.5% compared with that in CK, Pn tended to decrease with increasing EBR concentrations. Nonetheless, the Pn at the highest concentration of EBR (1.0 ppm) still remained significantly higher than that in CK. These results suggest that except for 0.01 ppm, rest of the concentrations of EBR showed a positive stimulatory effect on photosynthesis in tea plants.

### Effect of EBR on Summer Tea Yield

To assess the effect of EBR on growth and biomass production in tea plants, we measured the length of tea sprout, density of tea bud, weight of 100 buds, and summer tea yield (**Table 1**). Length of sprout increased with 0.1–1.0 ppm EBR, while density of bud increased with 0.05–1.0 ppm EBR. Likewise, weight of 100 buds showed an increased value under 0.05–1.0 ppm EBR treatment, where 0.1 ppm had the greatest effect. Similar to the trends in density of bud, yield of summer tea gradually increased with increasing concentration of EBR, resulting in the highest yield at 0.1 ppm EBR (increased by 37.79% compared with the CK). However, further increment in EBR concentration (0.5–1.0 ppm) slightly decreased the yield as compared with that in 0.1 ppm EBR.

**Table 1:** Effects of 24-epibrassinolide (EBR) on the growth and yield of summer tea.

EBR concentration (ppm)	Length of sprout (cm)	Density of bud (bud/m^2^)	Weight of 100 buds (g)	Yield (g/m^2^)
0	1.96 ± 0.105b	1701 ± 51.8d	9.30 ± 0.681c	260.7 ± 18.78c
0.01	2.00 ± 0.119b	1716 ± 114.6cd	9.55 ± 0.901bc	286.0 ± 17.71bc
0.05	2.15 ± 0.146ab	1976 ± 135.6b	10.45 ± 0.717ab	309.1 ± 21.00b
0.1	2.28 ± 0.130a	2274 ± 163.5a	11.07 ± 0.940 a	359.2 ± 21.36a
0.5	2.22 ± 0.097a	2139 ± 155.3ab	10.62 ± 0.837ab	320.8 ± 23.82b
1	2.19 ± 0.128a	1850 ± 107.5bc	10.54 ± 0.552ab	292.0 ± 20.02bc

*Mean denoted by different letters indicate significant differences between the treatments (P < 0.05).*

### EBR Stimulates Tea Quality in a Concentration-Dependent Manner

**FIGURE 2:**
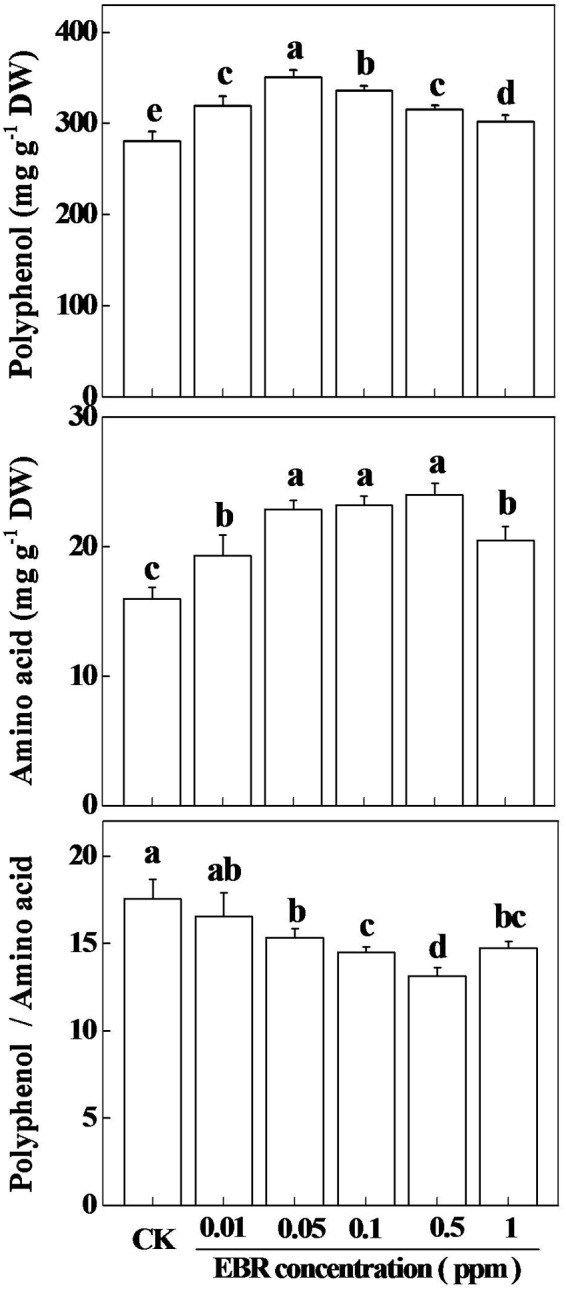
**Effect of different concentrations of 24-epibrassinolide (EBR) on total tea polyphenols (TP) and free amino acids (AA) concentrations in tea leaves.** Tea bushes were sprayed with various concentration of EBR (0, 0.01, 0.05, 0.1, 0.5, and 1.0 ppm) at 48 h prior to quantification of TP and AA. The results are expressed as the mean values ± SD, *n* = 6. Mean denoted by different letters indicate significant differences between the treatments (*P* < 0.05).

The composition of green tea especially TP, AA, and TP to AA ratio (TP/AA) are the major determinants of the quality of green tea (Liang et al., 1996; Tounekti et al., 2013). In our experimental tea garden, compared with spring tea, the concentration of TP in summer tea increased by 23%, while concentration of AA decreased by 31%, which resulted in a significantly increased TP to AA ratio (Supplementary Table S1). To assess whether exogenous EBR could alter the quality of green tea, we analyzed the concentrations of TP and AA, and the TP to AA ratio following application of various concentration of EBR. Results showed that all treated concentrations of EBR increased TP concentration in tea leaves, having highest increase (25.15%) at 0.05 ppm and lowest increase (7.74%) at 1.0 ppm EBR as compared with that in CK (**Figure 2**). Notably, TP gradually increased up to 0.05 ppm EBR, and then deceased with increasing concentration of EBR. Likewise, AA concentration increased with increasing concentration of exogenous EBR up to 0.05 ppm, and then remained more or less stable up to 0.5 ppm EBR. When EBR concentration was further increased, AA concentration decreased as compared with that at 0.5 ppm EBR. Thus, highest concentration of AA was recorded at 0.5 ppm EBR, which was increased by 50.20% compared with that in CK. Such alterations in TP and AA concentration, which were caused by exogenous EBR resulted in decreased TP to AA ratios, accounting for the lowest value at 0.5 ppm EBR. In quantitative figure, TP to AA ratio at 0.5 ppm EBR decreased by 25.13% compared with that in CK. All these results clearly indicated that effect of EBR on TP, AA, and TP to AA ratio was highly concentration dependent, where 0.5 ppm EBR showed the best effect by causing the highest reduction in TP to AA ratio, an important feature of quality green tea.

Next, we analyzed the concentrations of catechins, theanine, and caffeine in tea leaves following treatment with various concentrations of EBR (**Table 2**). Results showed that although all tested concentrations of EBR modulated catechins concentration, significant differences were noticed only at 0.05 and 0.1 ppm EBR. Theanine concentration was enhanced by 0.05–1.0 ppm EBR, where 0.5 ppm EBR caused the greatest increase (44.50%). However, the concentration of caffeine in tea leaves was not altered by the EBR treatment at any concentration (**Table 2**).

**Table 2:** Effects of 24-epibrassinolide (EBR) on the bioactive compounds in tea leaves relating to the quality of tea.

EBR concentration (ppm)	Catechins (mmol g^-1^ DW)	Theanine (mg g^-1^ DW)	Caffeine (mg g^-1^ DW)
0	154.7 ± 10.71c	8.27 ± 0.493d	39.41 ± 1.764a
0.01	165.2 ± 9.33bc	8.98 ± 0.518cd	40.62 ± 2.028a
0.05	178.7 ± 11.64ab	10.34 ± 0.466b	38.23 ± 1.575a
0.1	189.3 ± 13.24a	11.41 ± 0.725ab	40.44 ± 2.391a
0.5	171.6 ± 12.77abc	11.95 ± 0.937a	41.87 ± 2.726a
1	160.7 ± 13.53bc	9.77 ± 0.728bc	39.36 ± 1.311a

*Mean denoted by different letters indicate significant differences between the treatments (P < 0.05). DW, dry weight.*

### Time-Course Response of Tea Quality Parameters to Exogenous EBR

**FIGURE 3:**
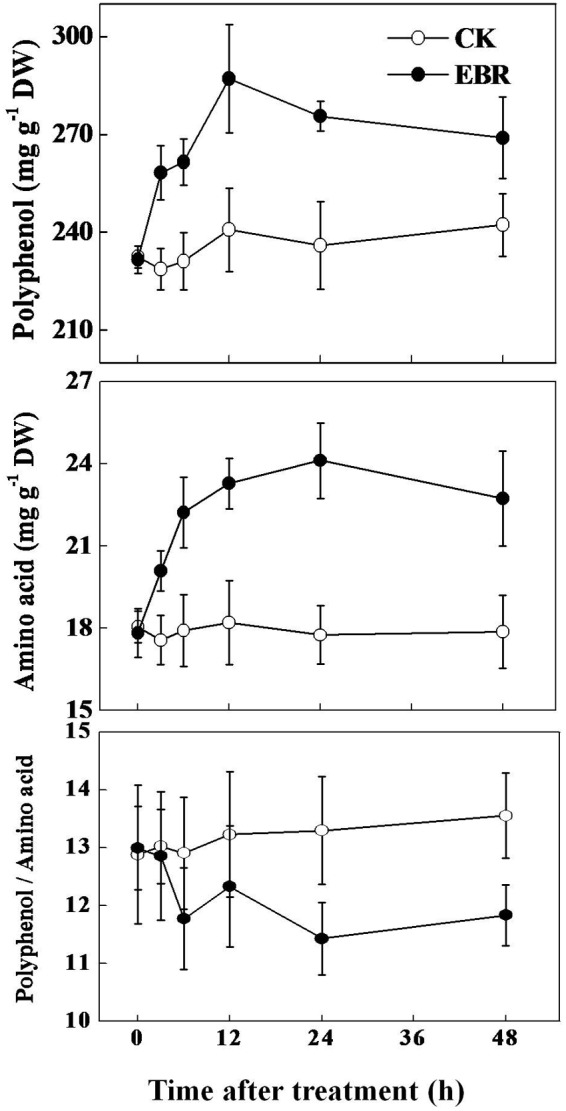
**Time-course effect of 24-epibrassinolide (EBR) on total tea polyphenols (TP) and free amino acids (AA) concentrations in tea leaves.** Tea bushes were sprayed with 0.5 ppm EBR. Measurements were taken at different time-points as mentioned in the respective figures. The results are expressed as the mean values ± SD, *n* = 6.

Based on TP to AA ratio data, we selected 0.5 ppm EBR to assess time-course response of TP and AA to exogenous EBR application. As shown in **Figure 3**, concentration of TP increased as early as 3 h after EBR application, reaching the maximum level at 12 h and then slightly decreased up to 48 h. Compared with the control treatment, EBR application increased TP concentration by 12.95, 13.13, 19.20, 16.78, and 11.01% after 3, 6, 12, 24, and 48 h, respectively. Similar to TP, concentration of AA also increased over time following EBR application. The concentration of AA increased by 14.41, 24.01, 27.85, 35.86, and 27.19% at 3, 6, 12, 24, and 48 h, respectively in EBR-treated tea leaves compared with that in CK. Importantly, such changes in TP or AA concentration by EBR application resulted in a remarkably decreased (by 14.04%) but stable TP to AA ratio at 24 h and onward. These results clearly suggest that EBR-induced improvement in tea quality is stable.

### Response of Secondary Metabolism-Related Enzymes and Genes to Exogenous EBR

**FIGURE 4:**
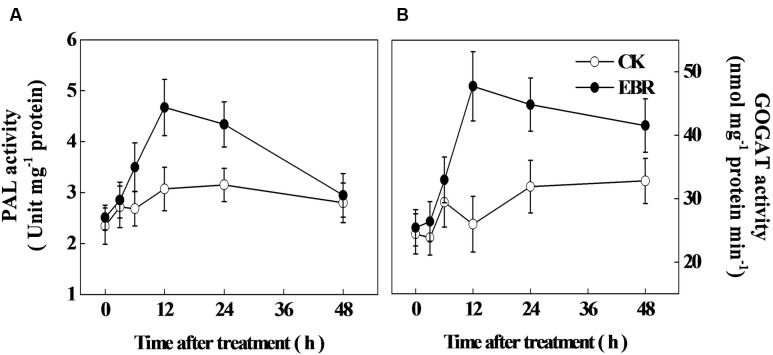
**Changes in the activity of (A) phenylalanine ammonia-lyase (PAL) and (B) glutamine: 2-oxoglutarate aminotransferase (GOGAT) as influenced by exogenous 24-epibrassinolide (EBR).** Leaf samples were harvested at indicated time-points following foliar spray of EBR. The results are expressed as the mean values ± SD, *n* = 6.

Next, we analyzed the activity of PAL, the first enzyme of phenylpropanoid pathway and the activity of GOGAT, a critical enzyme involved in theanine biosynthesis. In accord with time-course analysis of TP concentration, PAL activity increased gradually over time, reaching the peak at 12 h after EBR treatment (**Figure 4A**). Afterward, PAL activity declined and reached to the level of CK at 48 h. The activity GOGAT sharply increased at 12 h and remained higher up to 48 h after EBR treatment (**Figure 4B**).

To confirm whether the change in tea composition is attributed to a change in the biosynthesis of secondary metabolites, we analyzed the transcript levels of flavonoid biosynthetic genes *CsPAL* and theanine biosynthetic pathway-related genes *CsGS* and *CsGOGAT*. Transcript levels of all these genes were not much changed over time in CK leaves (**Figure 4**). However, in line with the TP concentration and PAL activity, transcript of *CsPAL* increased as early as 3 h, reaching the maximum level (1.3-fold) at 6 h after EBR treatment. Afterward, transcript levels of *CsPAL* gradually declined, but remained higher than that of CK even after 48 h. Transcripts of *CsGS* peaked at 6 h after EBR application and then gradually decreased up to 48 h. Notably, transcript of *CsGOGAT* reached maximum level (1.22-fold) at 12 h after EBR application, which was then slightly decreased but remained higher than that of CK treatment up to 48 h. Overall transcript data suggested that exogenous application of EBR stimulated transcriptional machinery causing accumulation of the highest levels of transcripts within early 6–12 h, which was then gradually declined, but remained higher than that in control treatment.

## Discussion

**FIGURE 5:**
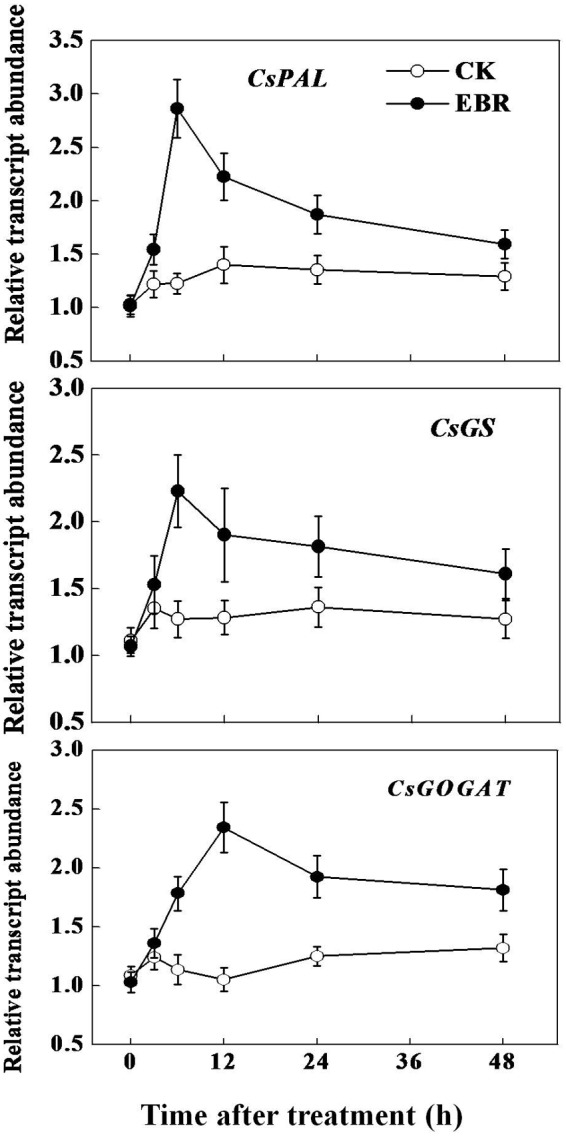
**Transcript levels of catechins and theanine biosynthetic genes as influenced by exogenous 24-epibrassinolide (EBR).** Leaf samples were harvested at indicated time-points following foliar spray of EBR. Transcript levels of genes were analyzed by qRT-PCR using gene-specific primer pairs (Supplementary Table S2).

Although green tea is often consumed considering its health benefits, its pleasant taste greatly influences overall consumption. For instance, a green tea that tastes bitter is not liked by the consumers, while a green tea that gives more brisk but less bitter taste is preferred by all (Xu et al., 2012). However, the production of quality green tea is greatly influenced by seasonal specificity (Tounekti et al., 2013). Summer days that are characterized by high temperatures have profound effect on the composition of green tea and thus TP to AA ratio in summer tea was significantly high compared with that in spring tea (Supplementary Table S1). In the current study, we showed that BRs, a well-known plant growth and stress hormone, could stimulate photosynthesis as well as secondary metabolism in tea plants during summer day, resulting in a decreased TP to AA ratio, a salient feature of quality green tea (**Figures 1** and **2**). Nonetheless, such response of tea plants to BR is dependent on the concentration of the exogenous EBR. Foliar application of EBR rapidly (as early as 3 h) induced transcript levels of key genes involved in the biosynthesis of catechins and theanine in tea leaves (**Figure 5**). Eventually such changes in transcript level resulted in a constant increment in the concentration of TP and AA but a decreased TP to AA ratio (**Figures 2** and **3**). These results suggest that BR might have potential to improve the quality of green tea beyond seasonal limitation.

Photosynthesis is the basic physiological process that provides both substrate and energy for the synthesis of primary metabolites, while secondary metabolites are subsequently derived from the primary metabolites (Verma and Shukla, 2015). In the current study, exogenous application of EBR increased net photosynthetic rate (Pn) in tea plants (**Figure 1**). The rate of photosynthesis is dependent on two main biochemical processes such as RuBisCO carboxylation and RuBP regeneration. Previous studies have shown that EBR increases photosynthesis in plants by increasing RuBisCO carboxylation rate and initial activity of RuBisCO (Yu et al., 2004; Jiang et al., 2012a). Therefore, it is plausible that EBR might also stimulate similar biochemical process to enhance photosynthesis in tea plants. It is well accepted that BR-induced photosynthetic responses are highly concentration dependent, where a moderate concentration stimulates photosynthesis, but a low or high concentration of EBR inhibits photosynthesis (Jiang et al., 2012a,b). However, the highest concentration that we used in the current study was much lower than 5 mM EBR which can decrease CO_2_ assimilation in cucumber (Jiang et al., 2012b). Therefore, each concentration (except for 0.01 ppm) of EBR significantly increased Pn in tea plants (**Figure 1**).

Several lines of evidence suggest that biosynthesis of secondary metabolites depends on various environmental cues (light, temperature, CO_2_, drought, salinity, ozone, UV-radiation) as well as endogenous signals (hormones and signaling molecules). Furthermore, change in only one factor can substantially alter the endogenous concentration of secondary metabolites even though other factors remain constant (Verma and Shukla, 2015). Similar to some other plant species such as tomato, grape, sweet basil and peppermint, exogenous application of EBR onto tea leaves remarkably stimulated secondary metabolism in the current study, which is evident by the significant increases in TP and catechins concentrations following foliar application of EBR (Ahammed et al., 2012; Xi et al., 2013; Koca and Karaman, 2015; Çoban and Göktürk Baydar, 2016). Notably, TP are synthesized through phenylpropanoid and flavonoid pathways, where PAL is the first enzyme that deaminated phenylalanine into cinnamic acid. *CsPAL* is the key gene that encodes PAL protein in tea. In our study, EBR induced transcript levels of *CsPAL* as early as 3 h after application, which was consistent with the enhanced concentration of TP at 3 h, indicating that EBR might stimulate the biosynthesis of TP by transcriptional regulation. Time-course analysis also revealed that EBR-induced changes in transcript levels resulted in a stable TP to AA ratio, which is sharply lower than that of CK, suggesting that EBR-mediated alteration in secondary metabolism is sustainable enough for improving green tea quality. However, our results argue with an earlier report concerning the effect of GA on TP in tea leaves, where GA remarkably decreased TP concentration (Liang et al., 1996). The discrepancy between two research findings could be due to difference in tea cultivars as well as kinds of plant hormones. It is worth mentioning that both BRs and polyphenols are synthesized from isopentenyl diphosphates that are provided by mevalonate pathway (Verma and Shukla, 2015). Therefore, BRs may function as a positive regulator of TP synthesis in plants.

In the current study, exogenous application of EBR increased AA as well as theanine levels in tea leaves (**Figure 2**; **Table 2**), which was in agreement with the effect of GA on AA concentration in tea leaves (Liang et al., 1996). It has been reported that BRs modulated biosynthesis of AA such as proline and glycine betaine in various plant species (Vardhini, 2014). However, to our knowledge, no literature is available regarding the effect of BR on free AA and theanine concentration in tea leaves. It is to be noted that theanine is the major tea AA accounting for more than 50% of total free AA in tea (Mu et al., 2015). Two enzymes, such as glutamine synthetase (GS) and GOGAT, which are considered as key determinants of theanine biosynthesis, catalyze the initial steps of NH_3_ assimilation into glutamic acid (Mu et al., 2015). To elucidate the mechanism underlying BR-induced AA accumulation, we analyzed transcript levels of key genes involved in theanine synthesis such as *GLUTAMINE SYNTHETASE (CsGS)* and *GLUTAMINE: 2-OXOGLUTARATE AMINOTRANSFERASE (CsGOGAT)*. As described in detailed under the Section “Results,” transcript levels of *CsGS* and *CsGOGAT* peaked as early as 6 and 12 h, respectively after foliar application of EBR, implying that EBR possibly promoted biosynthesis of theanine that largely contributed to the AA in tea leaves.

In conclusion, this study demonstrated that exogenous application of EBR not only promoted photosynthesis and yield, but also stimulated secondary metabolism in tea plants. EBR-mediated enhancement in secondary metabolism resulted in an increased TP and AA concentration but a decreased TP to AA ratio, which is considered as a desired parameter of quality green tea. In addition, EBR increased concentration of catechins and theanine without affecting concentration of caffeine in tea leaves. Further investigation at transcript levels revealed that EBR upregulated the expression of key genes involved in the biosynthesis of catechins and theanine. Our research sheds new light on the role of BR in the regulation of tea quality and thus it will pave the way for deciphering the precise role of BRs in tea secondary metabolism. It is known that BRs improve photosynthesis under high temperature stress in a range of plant species (Ahammed et al., 2014). As we carried out current experiment during summer days, when maximum mean temperature was approximately 37°C, it seems highly likely that EBR-induced enhancement in photosynthesis and secondary metabolism was associated with BR-mediated attenuation of heat stress in tea plants. Therefore, it will be interesting to further explore the role of BR in high temperature tolerance in tea plants.

## Author Contributions

XL and W-YH conceived and designed the research; XL, GA, Z-XL, LZ, J-PW, CS, PY, and L-PZ performed the experiments and analyzed the data; W-YH provided crucial reagents and supervised the study; XL and GA wrote the manuscript. All authors reviewed the manuscript.

## Conflict of Interest Statement

The authors declare that the research was conducted in the absence of any commercial or financial relationships that could be construed as a potential conflict of interest.
