# Supplementary material for: Brassinosteroids Improve Quality of Summer Tea (Camellia sinensis L.) by Balancing Biosynthesis of Polyphenols and Amino Acids
Source: Front Plant Sci. 2016 Aug 30;7:1304. doi: 10.3389/fpls.2016.01304 (PMC5003824; doi:10.3389/fpls.2016.01304)
Supplement: Supplementary file 1 [file Table_1.DOCX]

**Supplemental Table S1.** Comparison between spring tea and summer tea in the experimental tea garden of the Tea Research Institute, Chinese Academy of Agricultural Sciences, Hangzhou, China.

| Tea quality parameters  (mg g^-1^ DW) | Spring tea | Summer tea | Increase/decrease in summer tea |
| --- | --- | --- | --- |
| Tea polyphenols (TP) | 227.4±13.55 b | 280.0±10.77 a | 23.13 % |
| Free amino acids (AA) | 23.3±0.94 a | 16.0±0.90 b | -31.33 % |
| TP/AA | 9.76±1.26 b | 17.5±1.12 a | 79.30 % |

Mean denoted by different letters indicate significant differences at *P<0.05*. DW, Dry weight.

**Supplemental Table S2.** Primers used for real time RT-PCR assays

| **Gene** | **Encoding protein** | | **Accession No.** | **Primer pairs** |
| --- | --- | --- | --- | --- |
| *CsPAL* | [Phenylalanine ammonia-lyase](http://www.ncbi.nlm.nih.gov/protein/NP_001234126.1) | D26596 | | F: 5’-GAATGCCGGTCTTATCCACT-3’ |
|  |  |  |  | R: 5’-CGGTGAACACCTTGTCAAAC-3’ |
| *CsGS* | Glutamine synthetase | EU284131 | | F: 5’-GGAGGTTATCCTGGACCTCA-3’ |
|  |  |  |  | R: 5’-GGCAAGCCTTGTAGTGTGAA-3’ |
| *CsGOGAT* | Glutamine: 2-oxoglutarate | JN602371 | | F: 5’-ACACTGCCACATCTCAAAGG-3’ |
|  |  |  |  | R: 5’-CCAATTGATCAGCATTGACC-3’ |
| *Actin* |  | U60481.1 | | F: 5’-CTCAGCACATTCCAGCAGAT-3’ |
|  |  |  |  | R: 5’- ACTGCAGCTTTCCCAGAAAT-3’ |
